# Lateral atlantoaxial (C1-C2) joint steroid injections: a 22-year retrospective characterization of technique and clinical outcomes

**DOI:** 10.1093/pm/pnag017

**Published:** 2026-01-29

**Authors:** Kyril L Cole, Derrick K Wong, Troy A Hutchins, Lubdha M Shah, Miriam E Peckham

**Affiliations:** Department of Neurosurgery, University of Utah, Salt Lake City, UT, United States; Spencer Fox Eccles School of Medicine, University of Utah, Salt Lake City, UT, United States; Department of Radiology and Imaging Sciences, University of Utah Health Sciences Center, Salt Lake City, UT, United States; Department of Radiology and Imaging Sciences, University of Utah Health Sciences Center, Salt Lake City, UT, United States; Department of Radiology and Imaging Sciences, University of Utah Health Sciences Center, Salt Lake City, UT, United States

**Keywords:** C1-C2, lateral atlantoaxial joint injection, facet joint injection, epidural steroid injection, cervical fusion

## Abstract

**Background and purpose:**

Intra-articular injection of the lateral atlantoaxial joint is used for occipital pain; however, limited literature exists on technique demonstration, adverse event reporting, and real-world clinical outcomes. This study was designed to characterize procedural technique, describe clinical outcomes, and report subsequent cervical fusion rates following lateral atlantoaxial joint injections.

**Materials and methods:**

A retrospective cohort analysis was conducted on all lateral atlantoaxial joint injections performed at our institution from January 1, 2002, through August 1, 2024. Descriptive statistics characterized the cohort, procedural features, pain score changes, and cervical fusion rates during follow-up.

**Results:**

140 lateral atlantoaxial joint injections were performed on 104 patients (mean age 72.6 ± 12.4 years, 68.3% female), primarily by fluoroscopy (77.1%). Mean pre-injection pain was 5.99 ± 1.96, with immediate post-injection pain at 1.84 ± 2.33 and 1-month clinic visit pain at 4.28 ± 2.68. Significant reductions in pain from pre-injection were observed immediately post-injection (*P* < .0001) and at 1 month (*P* < .0001). Most injections were successful on the first attempt (97.1%), with no serious adverse events identified throughout follow-up documentation. Overall, 26.9% of patients underwent cervical fusion involving the C1-C2 level during available follow-up.

**Conclusion:**

This study comprises the largest cohort of patients with image-guided lateral atlantoaxial joint injections and provides descriptive data on technique, clinical outcomes, adverse events, and observed subsequent cervical fusion rates. Short-term reductions in pain were common 1 month from injections with no serious adverse events identified throughout follow-up and only one-quarter of patients undergoing eventual cervical fusion. This study adds valuable data to an uncommonly performed procedure.

## Teaser

This retrospective study describes a 22-year institutional experience with image-guided lateral atlantoaxial (C1-C2) joint injections, focusing on procedural technique, pain score changes, adverse events, and subsequent cervical fusion rates observed during follow-up. Short-term reductions in patient-reported pain were commonly observed, and no serious complications were identified. These findings provide descriptive, real-world context for an uncommon but clinically relevant spine intervention.

## Introduction

Lateral atlantoaxial (C1-C2) joint degeneration is a common cause of cervicogenic headaches and upper cervical pain, often presenting with a decreased range of motion and significant functional impairment.^1^ This condition frequently arises from degenerative arthritis, inflammatory disorders, or post-traumatic changes, which lead to referred pain mediated by the C2 nerve root and surrounding structures.^2^ Symptomatic cervical arthropathy has been estimated to affect approximately 13.7% of the adult population.^3^ Specifically for the C1-C2 joint, existing research indicates that atlantoaxial osteoarthritis-related morbidity affects 4%-5% of the population but is likely underdiagnosed and underrecognized as a significant source of neck pain. When conservative treatments such as physical therapy or anti-inflammatory medications fail to provide relief, intra-articular steroid injections have been successfully utilized as a minimally invasive therapeutic option,^1^^,^^4–6^ particularly in elderly individuals.^7^

Lateral atlantoaxial joint steroid injections aim to reduce inflammation, alleviate pain, and increase the range of motion where affected, with the added benefit of potentially delaying or avoiding more invasive surgical options, such as cervical fusion.^4^ However, these injections are often considered technically challenging due to the proximity of vital structures, including the vertebral artery, C2 dorsal root ganglion, and cervical spinal cord.^1^^,^^2^^,^^8^ Consequently, concern regarding procedural safety and inconsistent reports of efficacy have limited widespread adoption, with some arguing for abandoning the procedure altogether—citing risks of vascular injury due to variability in vascular anatomy, cerebral infarction, and procedural variability as major concerns, and transient adverse events cited as high as 18.5% in some cases.^4^^,^^9–11^ Despite these concerns, lateral atlantoaxial injections may be performed using appropriate image guidance and an understanding of at-risk anatomy.^1^^,^^12^ Additionally, limited literature exists evaluating long-term clinical outcomes or describing factors associated with subsequent cervical fusion after these injections.

We present a 22-year retrospective cohort analysis of all patients receiving lateral atlantloaxial joint injections at our institution. Our objectives were to describe procedural technique, characterize changes in pain scores, and report cervical fusion rates observed during follow-up. We additionally addressed procedural details and adverse events documented in our cohort. By addressing these objectives, we aim to provide clinicians with a clearer understanding of the real-world use and clinical course of patients undergoing lateral atlantoaxial injections.

## Methods

Data collection was conducted retrospectively with local IRB approval (IRB#00184838), and informed consent was waived for this retrospective cohort study. The STROBE guidelines were utilized in the manuscript’s preparation.

### Subjects, study design, and data collection

This retrospective single-center cohort study included all patients treated at our institution with lateral atlantoaxial joint injections over a 22-year period (January 1, 2002, through August 1, 2024). Our institution’s records were queried using “C1-C2 Facet” to capture our initial patient list. Results were filtered by exam description, including “CT guidance needle placement,” “IR cervical spine injection,” and “FL (or IR) guidance spinal injection.” Data for each case, including accession number, patient sex, patient age, and report text, were exported into a Microsoft Excel Spreadsheet (Microsoft Corporation, Redmond, Washington). As only patients with lateral atlantoaxial injections were queried and filtered, the analysis did not include a control group or any patients who did not receive injections. Patients receiving bilateral injections were included regardless of timing—whether performed concurrently or with an interval between procedures. No specific exclusion criteria were applied.

Subsequently, a search was conducted in the Picture Archiving and Communication System (PACS) using the patient’s accession numbers to record their corresponding demographic information (age at presentation, BMI, sex), level of intervention, needle gauge used, laterality of injection(s), number of attempts, modality used, pre-procedural imaging, successful injection, medication injected, and reported complications/side effects, as applicable. Additionally, pre- and post-procedure pain scores (including immediately post-procedure, 1-month post-procedure, and >1-month post-procedure) were recorded as available by directly asking patients at each timepoint to quantify their present level of pain (0-10, 10 being worst pain). Specific durations of pain were not recorded, and the cohort was not stratified based on pain pattern. For each atlantoaxial injection, electronic medical records were reviewed to identify any subsequent cervical spine fusion that included the C1-C2 level within 1 year following the injection. In patients with multiple injections, a separate 1-year observation window was applied to each injection. Operative reports, clinic notes, and imaging records were reviewed to verify whether a fusion involved the C1-C2 level.

### Statistical analysis

Descriptive statistics were reported for all baseline and outcome patient characteristics. Continuous variables were shown as mean±SD, while categorical variables were shown as *N* (%). Mean differences ± SD were calculated to assess changes in pain score from pre-injection, with paired *t*-tests to assess statistical significance. Statistical significance was determined as *P* < .05 in all analyses. Missing data were confirmed to be <10% for all studied variables, with missing data excluded from data analysis. Variables with any incomplete or missing data were specified in each respective table (eg, pre-procedure and post-procedure pain scores in Table 1). All analyses were performed using SPSS v.27 (IBM Corporation, Armonk, NY).

**Table 1:** Demographics of patients treated with lateral atlantoaxial injections for C1-C2 joint pathology.^a^

Variable	Patients treated (*N* = 104)
**Age, years**	72.6 ± 12.4
**Sex**	
Male	33 (31.7%)
Female	71 (68.3%)
**BMI**	25.8 ± 5.2
**Previous major elective surgery** ^b^	54 (51.9%)
**Average pain (scaled 0-10)**	
Immediate pre-injection pain	5.99 ± 1.96
Immediate post-injection pain	1.84 ± 2.33
1-month clinic f/u pain	4.28 ± 2.68
>1-month clinic f/u pain	5.90 ± 1.97
Median time between initial facet injection and last clinic follow-up visit, days	43.5 (IQR 33.5, 78.0)
**Received >1 injection procedures**	31 (29.8%)
Average number of injection procedures received	1.35±0.67
**Received cervical fusion surgery for pain**	28 (26.9%)
Median time between initial facet injection and surgical fusion, days	83 (IQR 55, 133)

Continuous variables as mean±SD.

Categorical variables as *N* (%).

a Missing data: 8/140 (5.7%) pre-operative pain scores, 9/140 (6.4%) post-operative pains scores. Follow-up pain scores limited to patients returning to clinic and documentation on clinic reports (absent for 32 of 140 injection visits (22.9%)). All other variables studied included complete data.

b Major elective surgery includes surgeries such as total hip arthroplasty, total knee arthroplasty, lumbar spine multilevel fusion, single level laminotomy, etc. Excludes minor elective procedures (eg, colonoscopy, cataract surgery, etc.).

Abbreviations: BMI, body mass index; C1, first cervical vertebra; C2, second cervical vertebra.

### Procedural technique

All cases were performed by 1 of 3 operators with 2-15 years of procedural experience during the 22-year search period with procedural consistency between operators developed through training and communication within the practice.

**Figure 1:**
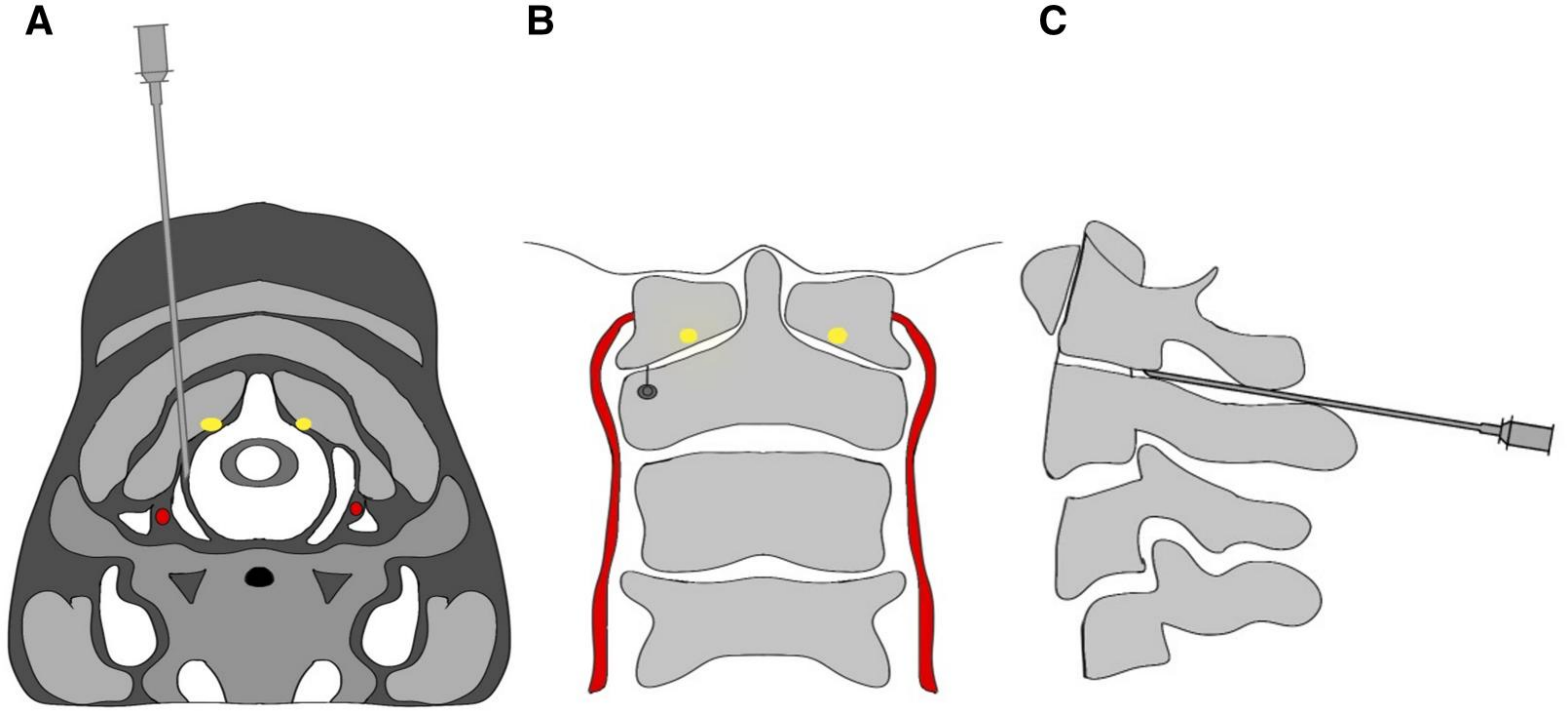
Line drawings depicting important anatomic structures to be aware of for safe placement of a needle within the lateral atlantoaxial joint by CT (A) and fluoroscopic (B, C) guidance. The vertebral artery (red) is noted just lateral to the joint, and the C2 dorsal root ganglion (yellow) lies more medial, directly posterior to the lateral mass. The course of the needle should avoid both of these structures, targeting the posterior and lateral third of the joint.

**Figure 2:**
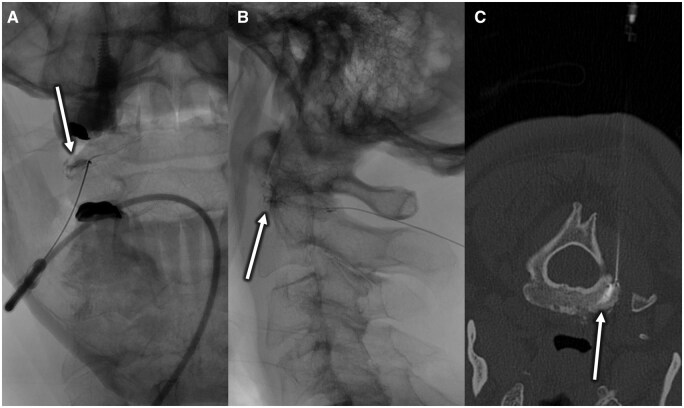
AP (A) and lateral (B) fluoroscopic views demonstrating placement of a needle within the lateral third of the left lateral atlantoaxial joint (patient lying prone) with contrast noted filling the joint space (arrows). Axial CT-guided (C) intraarticular placement of a 25-gauge needle into the lateral atlantoaxial joint with contrast filling the joint space (arrow).

Pre-procedure cross-sectional imaging was reviewed for each patient to determine the precise location of each facet joint and the surrounding at-risk anatomy. Prior to needle placement, patients were placed prone with a pillow placed under the chest to assist in neck flexion. Sterile technique was utilized to prepare the overlying skin site and anesthetized locally with 1%-2% lidocaine. Per patient preference, conscious sedation was used with IV Versed and Fentanyl and titrated to effect under continuous physiologic monitoring. Lateral scout CT or fluoroscopic images were obtained to determine the adequacy of position and neck flexion for joint accessibility. AP and lateral fluoroscopy, or small-view CT scan, were then performed to visualize the lateral atlantoaxial joint to determine the appropriate needle trajectory and insertion site with the ideal target along the outside third of the joint space ensuring avoidance of the more lateral vertebral artery and more medial location of the C2 dorsal root ganglion which lies just behind the lateral mass (Figure 1). CT axial view was utilized to visualize the lateral atlantoaxial joint, the vertebral artery in the adjacent transverse foramen, the approximate location C2 dorsal root ganglion posterior to the lateral mass, and the targeted posterior aspect of the joint (Figure 1). Under fluoroscopy or CT fluoroscopy guidance, a 22- or 25-gauge needle was advanced (with one exceptional case using 20-gauge), with needle selection determined by physician assessment of patient anatomy and comfort. Several images were taken to allow minor adjustments as necessary before entering the posterior aspect of the joint into the joint space, with care to avoid the C2 dorsal root ganglion medially and vertebral artery laterally (Figures 1 and 2).^13^

Once in the joint space, approximately 0.5 mL of Isovue-M 200 or 300 contrast was slowly injected to confirm intraarticular positioning, specifically assessing for contrast flow into the joint space via live fluoroscopic or CT images (Figure 2). Once confirmed, the medication (1 mL of 0.25%-0.5% bupivacaine with 10 mg/mL dexamethasone) was administered, with successful injection involving flow of medication into the joint space. Because the total injectate volume, including contrast, was 2.5 mL and likely exceeded the joint’s capacity, some of the mixture may have extravasated into the adjacent soft tissues. The needle was removed upon procedure completion, and a sterile dressing was applied to the area.

Post-procedure monitoring included standard sedation recovery (30 min of observation, vitals per nursing, and advancement of diet) as applicable, with usual discharge home with a driver. Post-procedure instructions stated avoidance of strenuous activity for the remainder of the day.

## Results

### Study cohort

Over a 22-year period, 104 patients received 140 lateral atlantoaxial joint steroid/anesthetic injections. Patients treated were an average age of 72.6 ± 12.4 years old, 68.3% female, and had an average BMI of 25.8 ± 5.2 (Table 1). Nearly a third (29.8%) of patients received >1 lateral atlantoaxial facet joint injection throughout the study period (1.35 ± 0.67 injections per patient). Mean pain by NRS was as follows: 5.99 ± 1.96 immediately pre-injection, 1.84 ± 2.33 immediately post-injection, 4.28 ± 2.68 at 1-month clinic follow-up, and 5.90 ± 1.97 after 1-month clinic follow-up (Table 1). The median cohort post-procedure follow-up time was 43.5 (IQR 33.5, 78) days. Eventually, 26.9% of patients received cervical fusion surgery over a median of 83 (IQR 53, 133) days from the time of their initial facet joint injection (Table 1).

### Imaging and injection-specific outcomes

Pre-procedural imaging most commonly included bone SPECT/CT (27.1%) or MRI (28.6%) for joint assessment (Table 2). Among the 140 injections performed, fluoroscopy was the primary modality utilized (77.1%), with most procedures on the left side (47.9%,14.3% bilateral). A 25-gauge needle was used for most injections (64.3%), with most requiring only one attempt for targeted injection (97.1%) and 95.7% being successful (Table 2). Reported side effects from the injections were endorsed in 7 (5.0%) patients, which included transient neck pain lasting <1 h, diffuse throat swelling lasting 1 h, one subjective report of a “drifting sensation” for 30 min, and transient dizziness for 10-15 min. No major adverse events were reported.

**Table 2:** Injection-specific variables.^a^

Variable	Injections performed (*N* = 140)
**Pre-procedural imaging used**	
Bone SPECT alone	38 (27.1%)
Bone SPECT + Other (CTA, MRI, or XR)	15 (10.7%)
CT alone	16 (11.4%)
CT + Other (MRI or XR)	21 (15.0%)
MRI alone	40 (28.6%)
MRI+ Other (CT or XR)	9 (6.5%)
**Modality used for image-guidance**	
CT	32 (22.9%)
Fluoroscopy	108 (77.1%)
**Side injected**	
Right	53 (37.9%)
Left	67 (47.9%)
Bilateral	20 (14.3%)
**Gauge of needle used**	
20	1 (0.7%)
22	48 (34.3%)
25	90 (64.3%)
**Number of attempts**	
Single attempt	136 (97.1%)
More than one needed	2 (1.4%)
**Reported side effects** ^b^	7 (5.0%)
**Success vs aborted procedures** ^b^	134 (95.7%)/6 (4.3%)

a One hundred forty injections performed across 104 patients.

b Side effects endorsed included acute suboccipital neck pain for 1 h, diffuse swelling and fullness within patient’s throat lasting 30 min, drifting sensation for 30 min, and transient dizziness for 10-15 min.

c Reasoning for aborted procedures was secondary to patient discomfort/pain in all cases.

Abbreviations: CT, computed tomography; CTA, computed tomography angiography; MRI, magnetic resonance imaging; SPECT, single photon emission computed tomography; XR, X-ray.

### Pain scores throughout follow-up

**Figure 3:**
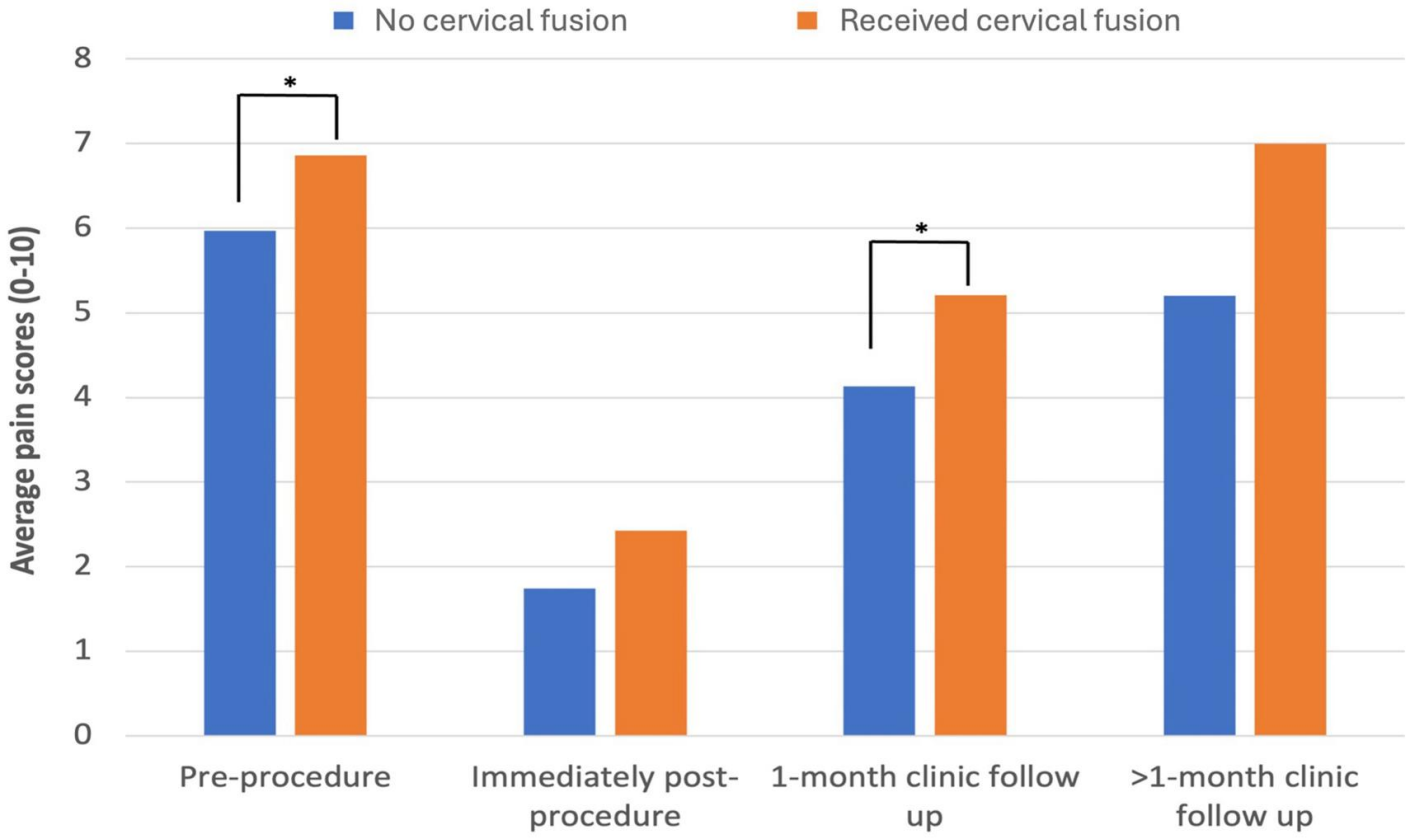
Average pain scores (scored 0-10; 10 being the worst) among patients receiving lateral atlantoaxial joint injections throughout the study period, assessing average pain differences among patients who did (orange) and did not (blue) receive eventual cervical fusion surgery. Pain assessed pre-injection, immediately post-injection, at 1-month clinic follow-up, and at any point after initial clinic follow-up visit.

Differences in pain score were analyzed from pre-injection, specifically for post-injection and 1-month follow-up. Significant reductions in pain score were observed at post-injection (4.19 ± 2.50, *P* < .0001) and at 1 month (1.81 ± 2.58, *P* < .0001). Change in pain score > 1 month was not analyzed due to low sample size (*N* = 10). Additionally, comparisons were made between those who eventually received cervical fusion and those who did not, assessing their peri-procedure pain scoring to understand its clinical relevance to our outcome of interest. On average, patients who did not receive cervical fusion demonstrated lower average pre-procedure pain scores than those who did receive cervical fusion (5.97 ± 2.05 vs 6.86 ± 1.38, *P* = .029) (Figure 3). No differences were seen between groups’ pain scores immediately post-injection (*P* = .122). Pain scores among patients who eventually received cervical fusion were higher on average at 1-month follow-up (*P* = .044), while pain scores at clinic visits over 1 month from injection date were not different between groups (*P* = .571) (Figure 3).

## Discussion

This study represents the largest known cohort to date describing atlantoaxial facet joint injections over a 22-year period. In this retrospective series, most patients demonstrated meaningful reduction in reported pain scores following injection, with 73.1% of our cohort not undergoing cervical spine fusion involving the C1-C2 level in available follow-up records. No major acute complications were identified in procedural or follow-up documentation. These findings contribute to the limited literature describing real-world use, technical considerations, and clinical outcomes of atlantoaxial facet injections, offering clinicians additional context when evaluating and performing this relatively uncommon procedure.

### Impact on pain relief

Evidence for intraarticular steroid injections across the cervical and lumbar spine has been mixed in prior trials.^14^ For the atlantoaxial joint specifically, available studies are limited and primarily retrospective in nature.^14^^,^^15^ Existing literature provides only modest support for the potential effectiveness of intraarticular steroid injections for short-term pain relief.^15–17^ To date, only one randomized study has been performed in the atlantoaxial joint on patients with rheumatoid arthritis, which demonstrated greater short-term improvement in pain and physical function in those receiving steroid compared with control.^18^

Within the context of limited evidence, our retrospective cohort showed notable reductions in patient-reported pain following the procedure, from an average of 6/10 pre-procedure to 2/10 immediately post-procedure and 4/10 at the standard 1-month clinic follow-up. Because many atlantoaxial injections are performed with sedative and/or opioid medications, we acknowledge that the immediate post-procedure reduction likely reflects both procedural and pharmacologic effects. Consequently, the 1-month follow-up scores represent a more meaningful estimate of the patient’s clinical response, demonstrating an average decrease of approximately 2 points on the 10-point scale. This general pattern was similar when stratified by eventual surgical fusion status (Figure 2), although patients who later underwent fusion tended to report higher baseline and 1-month post-injection pain scores. Such comparative trends seen help contextualize the variability in patient trajectories after lateral atlantoaxial injections and illustrate the range of symptomatic response encountered in real-world practice.

Notably, approximately 30% of patients underwent repeat injections during the study period. Although our design does not allow determination of patient preference or clinical decision-making, this pattern may reflect ongoing symptom recurrence and the real-world use of repeat injections as part of longitudinal management in select patients.

### Observed cervical fusion rates during follow-up

To our knowledge, prior studies evaluating lateral atlantoaxial joint injections have not described the subsequent cervical fusion rates observed in treated patients during follow-up. In our cohort, roughly one-quarter (26.9%) of patients underwent cervical fusion involving the C1-C2 level within the available follow-up period. These descriptive findings delineate the estimated proportion of patients who ultimately proceed to cervical fusion after receiving lateral atlantoaxial injections, offering clinicians useful context for how these procedures may be incorporated into real-world spine care trajectories.

Avoiding cervical fusion, when feasible, remains clinically important because of the procedure’s potential impact on cervical mobility, postoperative risks, and the development of adjacent segment disease, which can contribute to long-term functional limitations.^19^ Prior studies—including those by Radiee et al. and Holly et al.—have detailed these postoperative challenges and complication profiles associated with posterior cervical fusion, underscoring why understanding nonsurgical management patterns for upper cervical pain remains relevant.^20^^,^^21^

### Fluoroscopy vs CT-guided technique

To our knowledge, this is the first study to comprehensively describe the CT-guided technique for lateral atlantoaxial facet joint injections, as described in our methods. While fluoroscopy has traditionally been the standard imaging modality due to its real-time visualization and widespread availability, CT guidance offers superior anatomical detail, which may enhance procedural accuracy and safety, particularly for riskier, higher-level cervical injections than typically accessed by fluoroscopy, such as the atlantoaxial facet joint injections as is the focus of this study.^22–25^ Prior studies, such as Dietrich et al.,^26^ have demonstrated no significant differences in outcomes between CT or fluoroscopy-guided facet joint injections of the lumbar spine, though they noted higher radiation exposure in fluoroscopy-guided injections for physicians. Similar results between modalities in our study are in line with prior studies, emphasizing clinicians’ flexibility in selecting imaging modalities based on patient anatomy, operator preference, and resource availability.

### Lateral atlantoaxial steroid injection—safety considerations

Concerns regarding the safety of steroid injections in the high-risk anatomy of the C1-C2 region—including the vertebral artery, C2 dorsal root ganglion, and cervical spinal cord—have been well-documented. Risks such as vascular injury due to anatomical variability, cerebral infarction, and procedural inconsistencies between clinicians have likely contributed to ongoing hesitancy in adopting these injections.^1^^,^^2^^,^^4^^,^^9–11^

Aiudi et al. conducted the largest review of lateral atlantoaxial injection outcomes to date, reporting an 18.5% adverse event (AE) rate among 72 patients, with most events limited to post-procedural pain and vascular contrast uptake during the procedure. They identified an association between higher pre-procedural pain scores and AE occurrence, but no persistent or serious adverse events were reported.^4^ Similarly, Liou et al.^27^ described transient non-vertebral artery contrast uptake in 7% of 40 patients, again without resulting neurologic symptoms or lasting morbidity.

In our cohort of 140 injections, no permanent or serious AEs were identified in procedural documentation or follow-up visits. Transient symptoms occurred in approximately 5% of patients and included short-lived increases in neck pain (<1 h), throat fullness, a brief “drifting sensation,” or transient dizziness. Although these findings are reassuring, they do not establish the procedure as “safe,” nor can the true rate of rare but catastrophic complications be determined from retrospective series of this size. Upper cervical spine procedures inherently carry risk, and even large series are unlikely to detect extremely uncommon but clinically significant events.

Rather than defining safety, our results add to the growing descriptive evidence that severe complications appear to be uncommon when the procedure is performed with meticulous technique, appropriate imaging guidance, and a detailed understanding of regional anatomy.^1^^,^^12^

## Limitations

This study has limitations inherent to its retrospective, single-center design, including limited generalizability and unavoidable selection bias from evaluating only patients who received atlantoaxial injections. Importantly, the lack of a control group precludes any assessment of the efficacy of lateral atlantoaxial injections on pain scores or subsequent surgical outcomes. Additionally, follow-up information is limited to chart review within our institution, with the potential of missing subsequent procedures and cervical fusions performed at outside hospitals. Evaluation of technical success was also limited to the proceduralists’ reports as well as the images they chose to save for the record which do not always depict each aspect of the procedure. Because of multiple unmeasured confounders and practice variability, causal relationships cannot be inferred, including effects on cervical fusion or adverse events. Prospective, multicenter studies with standardized outcome measures and complete follow-up are needed to more precisely define the clinical outcomes and risk profile of lateral atlantoaxial joint injections.

## Conclusion

In this 22-year retrospective cohort including 140 procedures in 104 patients, lateral atlantoaxial joint injections were associated with notable short-term reduction in patient-reported pain scores, with the majority of patients not undergoing cervical fusion during available follow-up. No permanent or serious adverse events were identified immediately post-procedure and throughout follow-up, adding support for the utility of this procedure with rare complication rates. These findings contribute descriptive evidence regarding technique, clinical outcomes, and real-world practice patterns for this uncommon procedure.
